# Delayed Liver Regeneration after Partial Hepatectomy in Aged Nos2 Knockout Mice

**DOI:** 10.22074/cellj.2016.4878

**Published:** 2017-02-22

**Authors:** Deming Li, Jun Li, Gaiping Wang, Yanli Qin, Zhipeng Niu, Ziwei Li, Cunshuan Xu

**Affiliations:** 1 Key Laboratory for Cell Differentiation Regulation, Xinxiang, China; 2 College of Life Science, Henan Normal University, Xinxiang, China

**Keywords:** Aged, Liver Regeneration, Proliferation, Apoptosis

## Abstract

**Objective:**

Patients over 60 years of age have higher mortality and morbidity after major liver resections. Nitric oxide (NO) derived from the catalytic activity of Nos2 plays a beneficial role in liver regeneration (LR) after partial hepatectomy (PH). In this experiment, we evaluated the effect of *Nos2* knockout (KO) on LR in aged mice after PH.

**Materials and Methods:**

In this experimental study, 52 two-year-old *Nos2* KO and 46 the same age wild-type (WT) C57BL/6J mice were subjected to 2/3 PH. Liver tissues were collected at 11 time points after PH. Mice survival ratio and liver coefficient (liver-weight/ body-weight) was calculated. Transcript and protein levels were estimated by reverse transcriptase-quantitative polymerase chain reaction (RT-qPCR) and Western blot, respectively.

**Results:**

The aged *Nos2* KO mice had lower survival ratio (P=0.039) and liver coefficient (P=0.002) at the termination phase. *Nos2* transcript level was obviously increased after PH in WT mice and undetected in the *Nos2* KO mice. During LR, the expression at the transcript level of *Cyclin D1, Cyclin A2* and *Cyclin B1* and protein expression level of proliferation marker Ki67 and proliferation-associated transcription factors JNK1, NF-kB and STAT3 were decreased or delayed. The expression of pro-apoptotic proteins, CASPASE3, CASPASE9 and BAX, was increased in the *Nos2* KO mice.

**Conclusion:**

Decreased survival ratio and impaired LR in aged *Nos2* KO mice is probably due to decreased liver cell proliferation and increased liver cell apoptosis.

## Introduction

Adult hepatocytes are usually dormant with less than 0.01% of them undergoing mitosis at normal conditions. When the liver is subjected to surgical resection or exposed to toxins or viral infections, a complex process of regeneration is triggered to ultimately restore the quality and function of the liver (1,2). Two-third partial hepatectomy (PH) in rodents is a classic model for studying liver regeneration (LR). 

In response to PH, the remnant liver tissue initiates a synchronized and orderly response to allow the remaining cells to proliferate until the liver mass is recovered. For mice, the LR process is completed within 7 to 10 days. The regeneration process is actually a compensatory hyperplasia of remnant liver tissue rather than the re-growth of a lost structure (3). In general, the LR process is divided into three phases of priming, proliferation and termination (4). It has been shown that some pro-inflammatory cytokines such as tumor necrosis factor-alpha (TNF-α) and IL-6 are rapidly released during LR (5,6). Meanwhile, several immediate-early genes such as *Jun* and *Fos*, and transcription factors such as NF-kB and STAT3 are activated to promote hepatocyte proliferation (7-9). Liver to body weight ratio is about 4.5% in rodents and approximately 2.5% in humans. Cell proliferation stops once liver mass reaches an appropriate ratio of total body mass (10).

It has been reported that nitric oxide (NO) is released immediately after PH by liver parenchymal and non-parenchymal cells (11- 13). This unstable, small, gaseous molecule functions by acting as an intra- or extra-cellular messenger (14). NO synthesis is catalysed by nitric oxide synthase (Nos). Hitherto, three isoforms of Nos have been found, namely Nos1 (nNos), Nos2 (iNos) and Nos3 (eNos), with each having different physiological functions. NO derived from Nos3 and Nos1 plays important roles in regulating systemic blood pressure and organ blood ﬂow, whereas NO derived from Nos2 functions on pathogen killing and inﬂammatory processes (14, 15). However, if produced in excess NO can be harmful to the tissue of interest. Indeed, depending on the type of the stimulus and the amount/duration of *Nos2* expression, NO can be either beneficial or harmful to liver. *Nos2* activation can prevent sepsis and inhibit apoptosis. However, when the inflammatory cascade is activated and oxidative damage occurs under hemorrhagic shock and ischemia-reperfusion injury, increased *Nos2* expression results in deleterious effects. The expression of *Nos2* is mainly regulated at the transcriptional level, independent of calcium (16). However, a recent study showed that the expression of *Nos2* can be regulated by calciummediated signaling in hepatocytes through a mechanism independent of calcineurin (17). Multiple studies have shown that Nos2 plays an important role in hepatocytes regeneration. Its expression can be elevated within 4-6 hours after PH, whereas decreased *Nos2* expression impairs liver regeneration with increased liver damage. Nos2-synthesized NO after PH facilitates antiapoptosis (12, 18-20) and angiogenesis (12) as well sensitizing hepatocytes to mitogenic actions (21).

Most organs undergo pathophysiologic changes with aging and a gradual loss of reserve capacity. However, liver function can be preserved quite well due to its strong regeneration capacity (22- 24). As human life expectancy has increased greatly, more and more elderly patients with liver disease need partial hepatic resection. It has been reported that patients over 60 years of age have higher mortality and morbidity after major liver hepatectomy (25). Senescence augments the expression of *Nos2* at transcript and protein levels (26, 27). Previous studies have mainly focused on the role of *Nos2* in LR in young mice (12, 18-20). This study was therefore designed to examine the effect of *Nos2* on LR in aged mice.

## Materials and Methods

### Animals and the partial hepatectomy model

In this experimental study, *Nos2* mutant and WTC57BL/6J mice were purchased from Shanghai Laboratory Animal Co. Ltd. The described previously (28). Animals were kept at the Center of the Experimental Animals of Henan Normal University according to standard experimental conditions of temperature at 23 ± 3˚C with humidity of 35 ± 5% under a 12 hours light-dark cycle. Mice freely had access to regular laboratory chow diet. Two-year-old *Nos2* mutant and WT mice underwent 70% liver resection as described by Mitchell and Willenbring (29); the abdominal cavity was opened after ether anesthesia, the left lobe and the middle lobe were removed when their roots were fastened and finally abdominal cavity was sutured. The sham operation (SO) had the same procedure but excluded liver lobe excision. Three mice in each group were intraperitoneally anesthetized by 1% pentobarbital sodium (15 ml/kg) and then sacriﬁced and weighed at designated times after PH. Next, the remnant liver lobes were removed, weighed and stored at -80˚C for further analysis. All animal handling conformed to the Animal Protection Law of China and animal ethics.

### Mice survival ratio and liver coefficient

Three mice underwent PH at each time point. A total of 52 Nos2 mutant and 46 WT mice were used. The survival ratio of Nos2 mutant and WT mice was calculated at the priming phase, the proliferation phase and the termination phase. Liver coefficient was calculated by liver-weight/ body-weight.

### RNA isolation and reverse transcriptasequantitative polymerase chain reaction

Total RNA was isolated from liver tissues by Trizol reagent (Dingguo, China). Total RNA (2 μg) was used to synthesize cDNA using a reverse transcription kit (Promega). Quantitative polymerase chain reaction (qPCR) was performed using SYBR Green (Invitrogen, USA) on a Rotor-Gene 3000 PCR system (Corbett Robotics, Australia). *β-actin* expression was used to normalize gene expression. Relative mRNA levels were measured by the means of the 2^-ΔΔCt^ method (30). The oligonucleotide primers used are given in Table 1.

### Western blot analysis

As described by Zhang et al. (31), protein level was examined by the standard Western blot protocol. Proteins extracted from liver tissue were separated by 10% Sodium dodecyl sulfatepolyacrylamide gel electrophoresis (SDS-PAGE) and transferred onto a polyvinylidene difluoride (PVDF) membrane (Millipore). Membranes were blocked with 5% non-fat dry milk and incubated with desired antibodies, and then incubated with ECL ultra-sensitive luminescent substrate for 3 to 5 minutes. Gray scale scan and protein content analysis was done by the GE ImageQuant LAS400mini software. The antibodies used for WB were Ki67, total/phospho-JNK1, total/phospho NF-kB1/2, total/phospho-STAT3, CASPASE3, CASPASE9, BCL_2_, BAX, and β-ACTIN, all of which were produced by Boaosen China Inc. (Beijing, China).

**Table 1:** Quantitative polymerase chain reaction (qPCR) primers and their annealing temperatures


Gene	Sequence primer (5´- 3´)	Annealing temperature

*Nos2*	F: TCCTACACCACACCAAAC	51˚C
	R: CTCCAATCTCTGCCTATCC	
*Cyclin D1*	F: TACCGCACAACGCACTTTCTT	60˚C
	R: GACCAGCCTCTTCCTCCACTT	
*Cyclin A2*	F: CCCCAGAAGTAGCAGAGTTTGT	60˚C
	R: AAGGTACGGGTCAGCATCTATC	
*Cyclin B1*	F: AAATACCTACAGGGTCGTGAAGTG	60˚C
	R: CATCTGTCTGATCTGGTGCTTAGTG	
*Fos*	F: GTTTCAACGCCGACTACGAG	60˚C
	R: TTGGCACTAGAGACGGACAGA	
*Jun*	F: CAGAGTTGCACTGAGTGTGGC	60˚C
	R: GCAGTTGGTGAGAAAATGAAGAC	
*Nf-kb1*	F: TGGAGGCATGTTCGGTAGTG	60˚C
	R: CCTGCGTTGGATTTCGTGA	
*Nf-kb2*	F: ATGGCACAGGACGAGAACG	60˚C
	R: AGGTGGTTGGTGAGGTTGATG	
*β-actin*	F: CCGTAAAGACCTCTATGCCAACA	60˚C
	R: CGGACTCATCGTACTCCTGCT	


### Statistical analysis

Data were expressed as mean ± standard error (SEM). Statistical differences between groups were examined using the independent-samples t test in SPSS 16.0 (SPSS Inc., Chicago, USA). P<0.05 was considered statistically signifi cant.

## Results

### Expression of *Nos2* during liver regeneration

**Fig.1:**
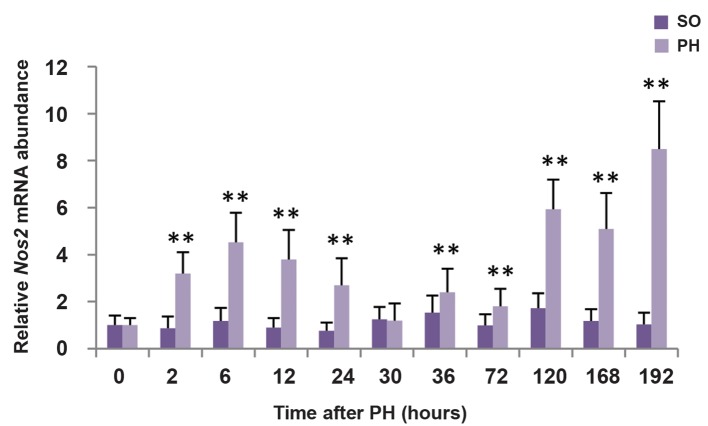
The expression of Nos2 gene in liver of WT mice after SO and PH. Transcript levels of Nos2 was determined by RT-qPCR. *β-actin* was used to normalize gene expression. Values are mean ± SEM (n=3, **; P<0.01 vs. SO group). SO; Sham operation, PH; Partial hepatectomy, WT; Wild-type, and RT-qPCR; Reverse transcription-quantitative polymerase chain reaction.

To determine the expression pattern of *Nos2* during the course of LR, RT-qPCR was performed in WT mice after PH or SO. Expression of *Nos2* mRNA was remarkably increased during regeneration process except at 30 hours (Fig .1, P<0.01 at 9 time points). Nos2 transcript was more abundant at the priming phase and the termination phase than the proliferation phase.

### Decreased survival ratio and liver regeneration in *Nos2* KO mice

**Fig.2:**
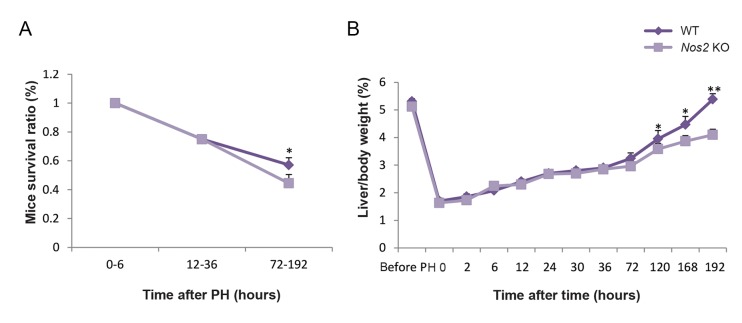
Mice survival ratio and liver/body weight ratio changes after PH. A. Mice survival ratio at the three regeneration phases (n=3-8, the priming phase: 0-6 hours; the proliferation phase: 12-36 hours and the termination phase: 72-192 hours) and B. Liver recovery after PH was determined by liver/body weight ratio at indicated time points. Data are mean ± SEM (n=3, *; P<0.05 and **; P<0.01 vs. WT group). PH; Partial hepatectomy, WT; Wild-type, and *Nos2* KO; *Nos2-/-*.

After PH, the survival ratio was decreased with time for both *Nos2* KO and WT mice. There was no significant difference between *Nos2* KO and WT mice at the priming phase and the proliferation phase. However, during the termination phase, the survival ratio was significantly lower in *Nos2* KO mice than WT mice (Fig .2A, 57.14% in WT mice and 44.44% in KO mice, P=0.039). There was no significant difference in liver coefficient within 72 hours after PH, however, the liver coefficient was lower in *Nos2* KO mice than WT mice from 120 hours to 192 hours after PH (Fig .2B, P=0.020, 0.047 and 0.002, respectively).

### Decreased expression of Cyclins and cell proliferation in *Nos2* KO mice

**Fig.3:**
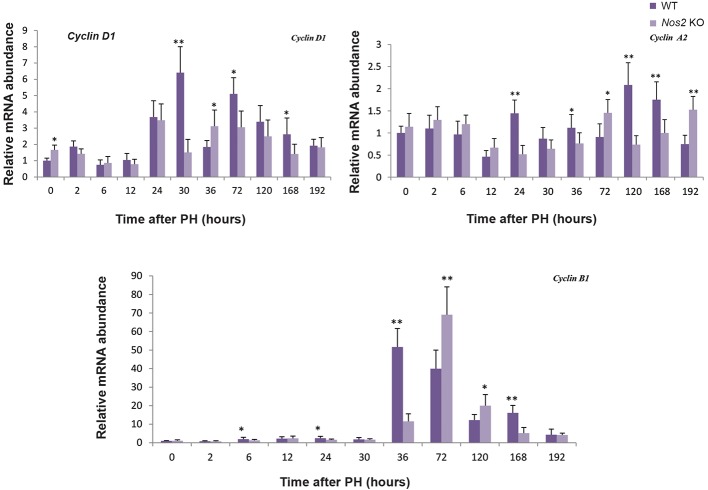
The liver expression of cyclin-related genes in WT and *Nos2* KO mice after PH. Expression of *Cyclin D1, Cyclin A2* and *Cyclin B1* after PH was measured by RT-qPCR and normalized with *β-actin* levels, serving as an internal control. The value from 0 hour time-point in WT mice was set as one-fold. Values are mean ± SEM (n=3, *; P<0.05 and **; P<0.01 vs. WT group). PH; Partial hepatectomy, WT; Wild-type, *Nos2* KO; *Nos2-/-*, and RT-qPCR; Reverse transcription-quantitative polymerase chain reaction.

**Fig.4:**
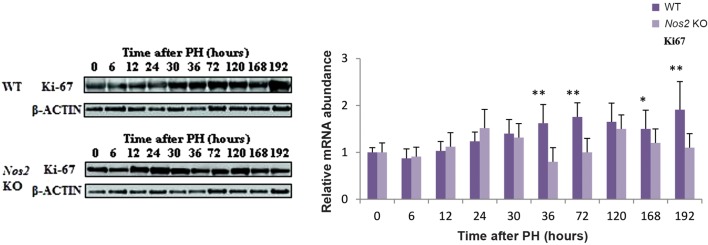
The expression of the proliferation-related protein Ki67 was evaluated by Western blotting analysis in the liver of *Nos2* KO and WT PH mice. Data are expressed as mean ± SEM (n = 3, *; P<0.05 and **; P<0.01 vs. WT group). PH; Partial hepatectomy, WT; Wild-type, and *Nos2* KO; Nos2-/-.

To evaluate proliferation of hepatocytes in response to PH, we undertook RT-qPCR analysis for cell-cycle associated genes *Cyclin D1, Cyclin A2* and *Cyclin B1*, and Western blot analysis for the proliferation marker, Ki67. During the early LR phase, the expression level of *Cyclin D1* and *Cyclin A2* was not significantly different between *Nos2* KO mice and WT mice. Compared with WT mice, the expression level of *Cyclin B1* was signifi cantly lower in the *Nos2* KO mice at 6 hours and 24 hours after PH (P<0.05). Furthermore, the expression of all three genes was delayed in the *Nos2* KO mice at the later phase (Fig .3, P<0.05 or P<0.01). Western blot analysis showed a lower expression of Ki67 in the *Nos2* KO mice compared with WT mice from 36 hours to 192 hours after PH (Fig .4, P<0.05 at 168 hours, P<0.01 at 36 hours, 72 hours and 192 hours).

### Evaluation of transcript expression of Fos and Jun as immediate early genes

**Fig.5:**
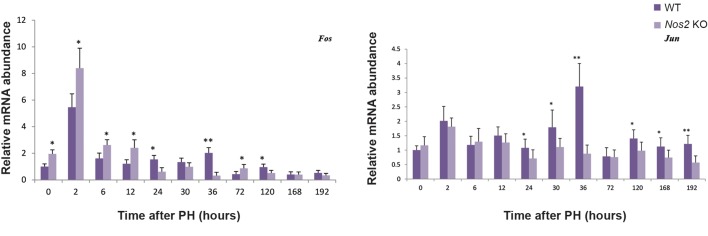
The expression of the immediate early genes after PH. Transcript levels of Fos and Jun were determined by RT-qPCR and normalized with *β-actin* (n=3, *; P<0.05 and **; P<0.01 vs. WT group). PH; Partial hepatectomy, WT; Wild-type, *Nos2* KO; *Nos2-/-*, and RT-qPCR; Reverse transcription-quantitative polymerase chain reaction.

RT-qPCR was undertaken to examine transcript expression of immediate early genes, *Fos* and *Jun*. The expression of *Fos* was higher at the early phase but lower at the later phase in the *Nos2* KO mice when compared with the WT mice. The expression of *Jun* was almost the same at the early phase, however, its expression decreased at the later phase in the *Nos2* KO mice (Fig .5, P<0.05 and P<0.01).

### Evaluation of expression of proinflammatory cytokines TNF-α and IL-6

**Fig.6:**
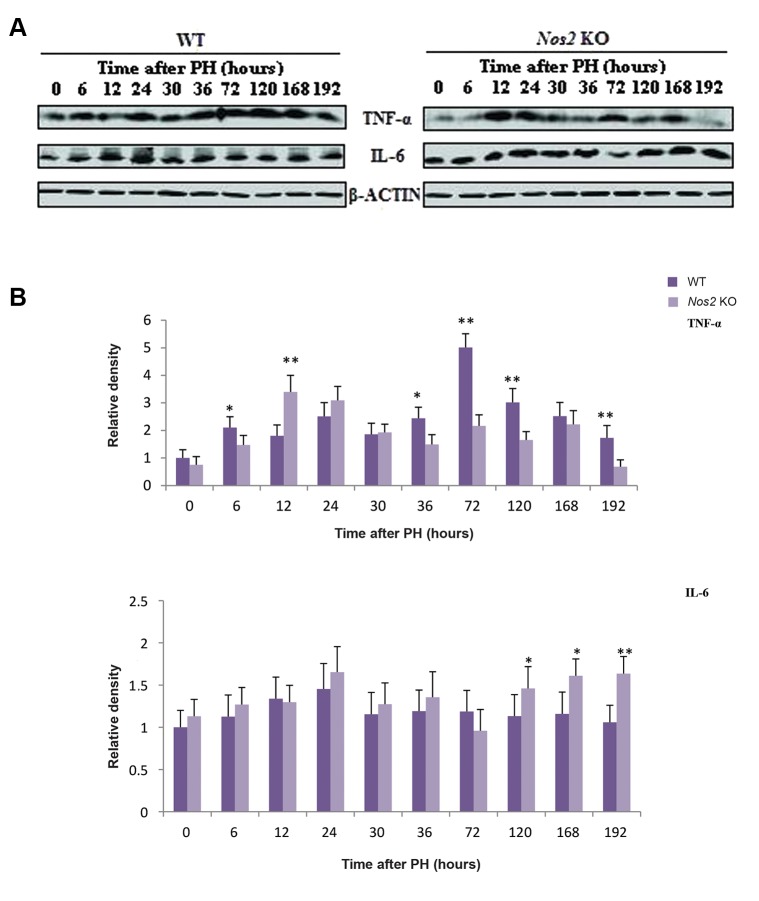
The expression of the proinflammatory cytokines TNF-α and IL-6 during LR. A. Western blot analysis of TNF-α and IL-6 expression and B. Densitometric analysis of the results shown in A. β-ACTIN was used as a loading control. Values are mean ± SEM (n=3, *; P<0.05 and **; P<0.01 vs. WT group). WT; Wild-type, *Nos2* KO; *Nos2-/-*, and LR; Liver regeneration.

Western blot analysis was performed to detect expression of TNF-α and IL-6 at the protein level. TNF-α was decreased and delayed during LR process in the *Nos2* KO mice compared with the control. The expression of IL-6 remained unchanged during the priming phase and the proliferation phase, however, its expression increased from 120 hours to 192 hours (Fig .6, P<0.05 and P<0.01).

### Alteration of proliferative and apoptotic signaling in *Nos2* KO mice

To understand the mechanism of the delayed LR in aged *Nos2* KO mice, we also examined the expression of proliferation- and apoptosis-associated genes by Western blot analysis.

Activation of JNK1 was delayed in *Nos2* KO aged mice compared with WT mice. The expression of NF-kB1 was decreased at most of the time points, however, the expression of NF-kB2 was almost unchanged except a few time points (Figes.7, 8, P<0.05 and P<0.01). STAT3 was also decreased at multiple time points (Fig .8, P<0.05 at 30 hours, 168 hours and 192 hours, P<0.01 at 120 hours).

The expression of pro-apoptotic executive protein CASPASE3 was raised almost at all time points in the *Nos2* KO mice when compared with controls. The expression of CASPASE9 was up-regulated at most of the time points. Although the expression of apoptosis-inhibiting protein BCL_2_ was increased throughout the LR course in the WT mice, it showed little change in the *Nos2* KO mice. The expression of apoptosis-promoting protein BAX was dramatically increased at 36 hours and at the termination phase in the *Nos2* KO mice, however, it had no significant change in the WT mice (Fig .9, P<0.05 and P<0.01).

**Fig.7:**
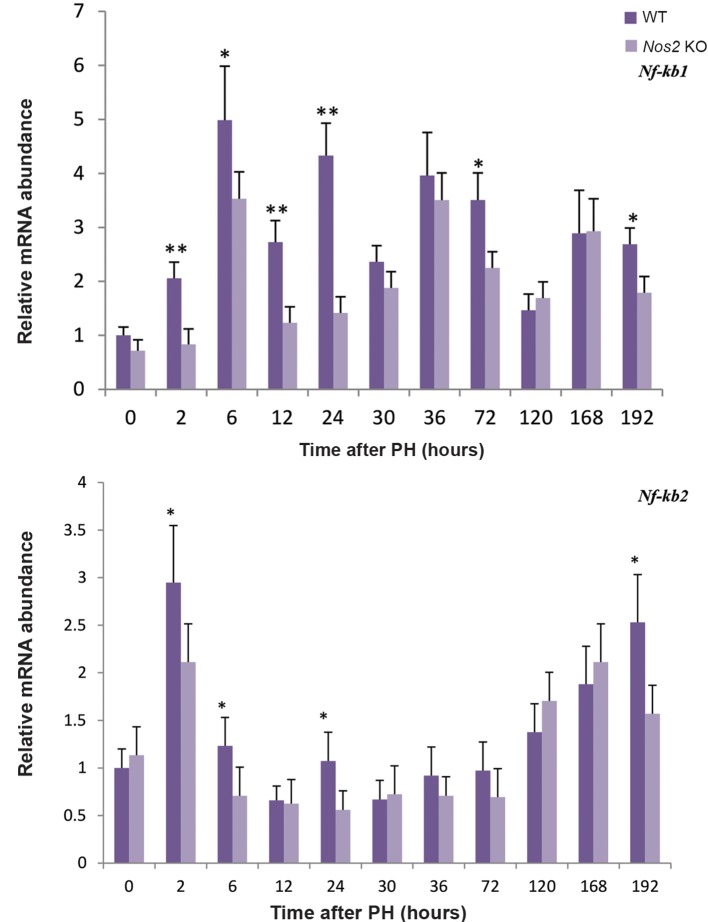
The expression of *Nf-kb* during LR. RT-qPCR analysis of Nf-kb transcript in the regenerating liver after PH. *β-actin* was used as a loading control. Values are mean ± SEM (n=3, *P<0.05; **P<0.01 vs. WT group). LR; Liver regeneration, PH; Partial hepatectomy, RT-qPCR; Reverse transcription-quantitative polymerase chain reaction, WT; Wild-type, and *Nos2* KO; *Nos2-/-*.

**Fig.8:**
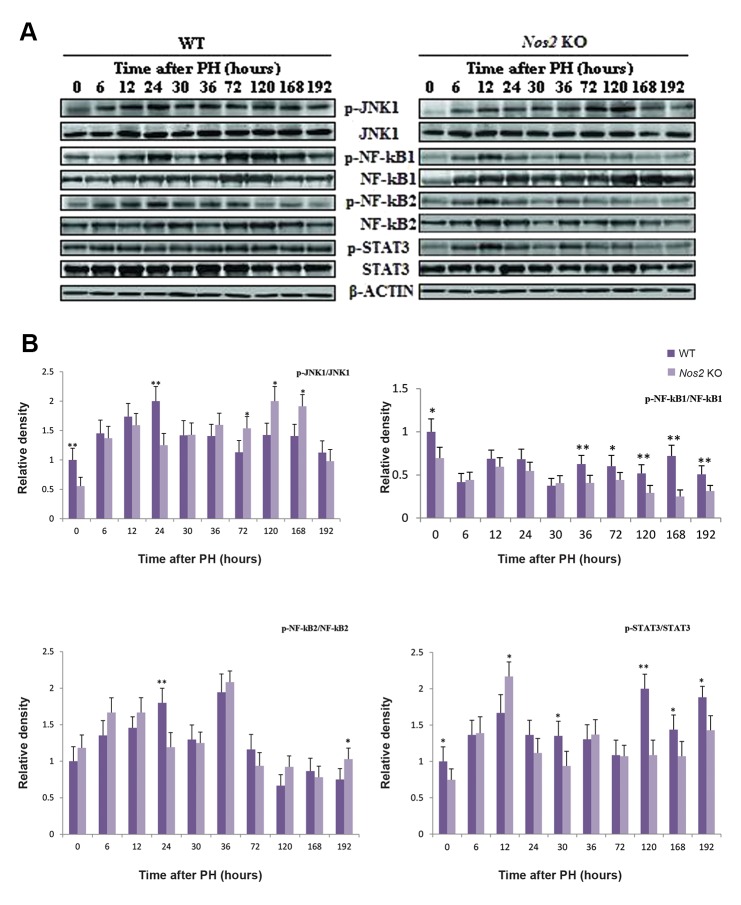
The expression of relevant transcription factors during LR. A. Western blot analysis of phospho/total-JNK1/NF-KB1/2 and phospho/ total-STAT3 and B. Densitometric analysis of the results shown in A. β-ACTIN was used as a loading control. Values are mean ± SEM (n=3, *; P<0.05 and **; P<0.01 vs. WT group). LR; Liver regeneration, WT; Wild-type, and *Nos2* KO; *Nos2-/-*.

**Fig.9:**
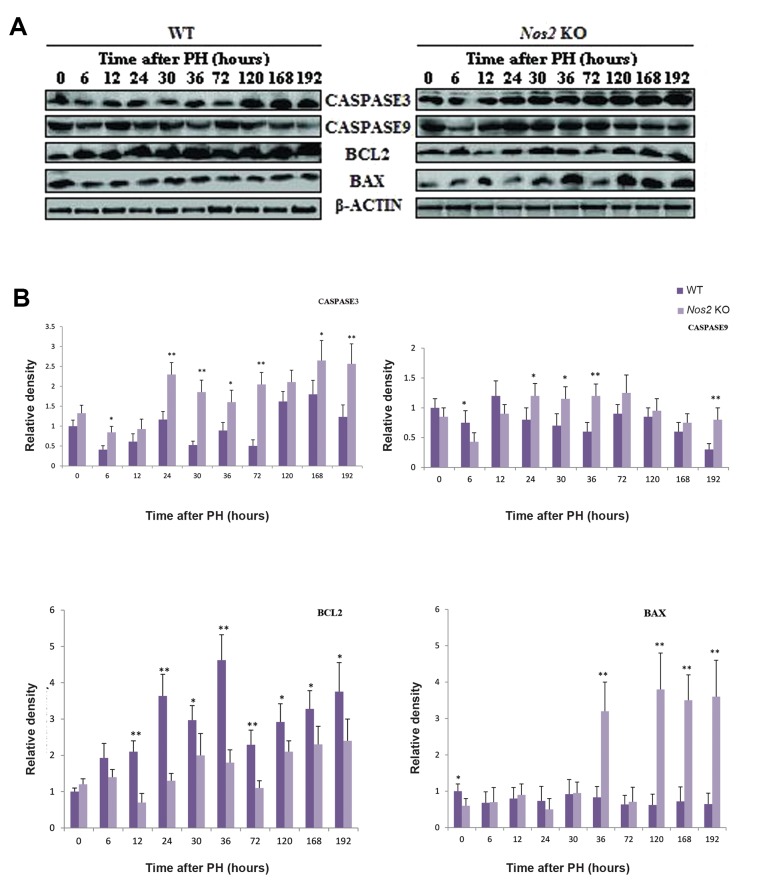
The expression of pro-apoptotic proteins during LR. A. Western blot analysis of CASPASE3, CASPASE9, BCL_2_, and BAX expression and B. Densitometric analysis of the results shown in A. β-ACTIN was used as a loading control. Values are mean ± SEM (n=3, *; P<0.05 and **; P<0.01 vs. WT group). LR; Liver regeneration, WT; Wild-type, and *Nos2* KO; *Nos2-/-*.

### Discussion

In the present study, we compared liver regeneration response between aged *Nos2* KO and WT mice. Although aged, both showed strong LR capability after 2/3 PH. Liver coefficient was recovered on the 8^th^ day after PH in the WT mice. However, this was not completely recovered at the same time point for the *Nos2* KO mice. At the termination phase, regeneration response was slower with lower survival ratio in the *Nos2* KO mice as compared with WT mice. Impaired LR in the aged *Nos2* KO mice was associated with decreased proliferation and increased apoptosis signals, indicating a key role in LR for *Nos2*. 

To better understand the LR reaction in the aged *Nos2* KO mice after PH, we examined the expression level of several cell cycle regulatory genes, such as *Cyclin D1*, marker of G1 phase, and *Cyclin A2*, regulator of the G1/S transition phase (32). When compared with WT mice, the expression of *Cyclin D1* and *Cyclin A2* showed significant down-regulation at several time points; furthermore, the expression of *Cyclin D1* and *Cyclin A2* was delayed in the KO mice at the later phase after PH. The expression of Cyclin B1, a key factor in G2/M transition during LR (33), was dramatically elevated at the later phase in both types of mice. However, the expression of *Cyclin B1* was delayed in the aged *Nos2* KO mice. These data suggested that the *Nos2* KO probably slowed hepatocyte cell cycle. The expression of the proliferation marker Ki67 was also decreased at the later phase of LR, suggesting impaired proliferation of hepatocytes in the aged *Nos2* KO mice. 

The immediate early genes *Jun* and *Fos* (34) and the inflammatory factors TNF-α and IL-6 (35,37) play important roles in the initiation and progression of LR. The expression of Fos transcripts was significantly elevated at the early phase in the aged *Nos2* KO mice, which probably compensated for the disadvantageous effect of Nos2 absence. The expression of both *Jun* and *Fos* was attenuated at the later phase in the aged *Nos2* KO mice. Activation of *Fos* and *Jun* can increase the transcriptional activity of genes involved in cell cycle progression (38). The reduction of *Fos* and *Jun* expression was synchronous with expression changes of cell cycle regulatory genes *Cyclin D1, Cyclin A2*, and *Cyclin B1*. 

Cell proliferation and apoptosis are modulated by some key signaling pathways. Protein expression of TNF-α was decreased and delayed in *Nos2* KO mice with downstream signaling molecule NF-kB strikingly decreased. NF-kB regulates the transcription of *Cyclin D1* (39). The weaker expression of NF-kB perhaps causes the decrease in the expression level of *Cyclin D1*. JNK activation has been shown to play a key role during LR. Reduced LR has been related to the attenuation of JNK activation, probably mainly JNK1, as JNK2 seems dispensable during LR (40,42). The activity of JNK1 contributes to the phosphorylation and activation of STAT3 (42,43). Compared with WT mice, the expression of IL-6 in *Nos2* KO mice remained unchanged during the early LR phase, however, the activation of JNK1 and IL-6 downstream signal, STAT3, was decreased and delayed, which probably resulted in impaired LR at the termination phase in the aged *Nos2* KO mice. In addition, the protein expression of pro-apoptotic genes CASPASE3, CASPASE9 and BAX were strikingly increased in *Nos2* KO mice. Although the expression level of BCL_2_ was unchanged in *Nos2* KO mice after PH, it was strongly expressed in WT mice. These results suggested that apoptosis was increased during the later LR phase in *Nos2* KO mice, thus resulting in less cell proliferation. A previous study found that impaired LR in young *Nos2* KO mice wasn’t due to impaired liver cell proliferation but due to increased liver cell apoptosis (16). Other studies also found inhibition of *Nos2* expression resulted in decreased DNA synthesis, delayed LR and changes resembling that of DNA ploidy (12,19,44). Over-expression of Nos2 has been shown to result in attenuated LR but also inhibits hepatocyte apoptosis (20). NO, a ubiquitous anti-apoptotic molecule, derived from Nos2 catalysis may protect liver by reducing the number of apoptotic liver cells. Higher mortality and attenuated LR in aged *Nos2* KO mice at the termination phase is likely to be due to increased apoptosis and decreased proliferation. Inhaling low concentrations of gaseous NO has been already in clinical application for the treatment of persistent pulmonary hypertension of the newborn (45) and may thus be used post-PH in patients over 60 years of age, who have a poor prognosis after major liver resections (25). Our findings also suggest that inhaling appropriate concentrations of NO may help to reduce mortality and morbidity in old patients suffering PH. 

## Conclusion

Decreased and delayed expression of *Cyclin D1, Cyclin A2* and *Cyclin B1* in aged *Nos2* KO mice probably retard the progression of hepatocytes into the cell cycle. Declined and retarded expression of proliferation-associated transcription factors JNK1, NF-kB and STAT3 can also reduce hepatocyte proliferation. Furthermore, attenuated expression of apoptosis inhibitory protein BCL_2_ and increased expression of pro-apoptotic proteins CASPASE3, CASPASE9 and BAX may lead to apoptosis. Together, these changes may result in the lower survival ratio and liver coefficient as observed for aged *Nos2* KO mice. 

## References

[1] Diehl AM, Rai R (1996). Review: regulation of liver regeneration by pro-inflammatory cytokines. J Gastroenterol Hepatol.

[2] Michalopoulos GK, DeFrances MC (1997). Liver regeneration. Science.

[3] Greene AK, Puder M (2003). Partial hepatectomy in the mouse: technique and perioperative management. J Invest Surg.

[4] Fausto N, Campbell JS, Riehle KJ (2006). Liver regeneration. Hepatology.

[5] Gallucci RM, Simeonova PP, Toriumi W, Luster MI (2000). TNF-alpha regulates transforming growth factor-alpha expression in regenerating murine liver and isolated hepatocytes. J Immunol.

[6] Clavien PA (1997). IL-6, a key cytokine in liver regeneration. Hepatology.

[7] Cressman DE, Greenbaum LE, Haber BA, Taub R (1994). Rapid activation of post-hepatectomy factor/nuclear factor kappa B in hepatocytes, a primary response in the regenerating liver. J Biol Chem.

[8] Cressman DE, Diamond RH, Taub R (1995). Rapid activation of the Stat3 transcription complex in liver regeneration. Hepatology.

[9] Costa RH, Kalinichenko VV, Holterman AX, Wang X (2003). Transcription factors in liver development, differentiation, and regeneration. Hepatology.

[10] Van Thiel DH, Gavaler JS, Kam I, Francavilla A, Polimeno L, Schade RR (1987). Rapid growth of an intact human liver transplanted into a recipient larger than the donor. Gastroenterology.

[11] Obolenskaya MYu, Vanin AF, Mordvintcev PI, Mülsch A, Decker K (1994). Epr evidence of nitric oxide production by the regenerating rat liver. Biochem Biophys Res Commun.

[12] Carnovale CE, Scapini C, Alvarez ML, Favre C, Monti J, Carrillo MC (2000). Nitric oxide release and enhancement of lipid peroxidation in regenerating rat liver. J Hepatol.

[13] Ronco MaT, de Luján Alvarez M, Monti JA, Carrillo MaC, Pisani GB, Lugano MaC, et al (2004). Role of nitric oxide increase on induced programmed cell death during early stages of rat liver regeneration. Biochim Biophys Acta.

[14] Muriel P (2000). Regulation of nitric oxide synthesis in the liver. J Appl Toxicol.

[15] Pacher P, Beckman JS, Liaudet L (2007). Nitric oxide and peroxynitrite in health and disease. Physiol Rev.

[16] Li J, Billiar TR (1999). Nitric Oxide.IV.Determinants of nitric oxide protection and toxicity in liver. Am J Physiol.

[17] Zhang B, Crankshaw W, Nesemeier R, Patel J, Nweze I, Lakshmanan J (2015). Calcium-mediated signaling and calmodulin-dependent kinase regulate hepatocyte-inducible nitric oxide synthase expression. J Surg Res.

[18] Rai RM, Lee FY, Rosen A, Yang SQ, Lin HZ, Koteish A (1998). Impaired liver regeneration in inducible nitric oxide synthasedeficient mice. Proc Natl Acad Sci USA.

[19] Zeini M, Hortelano S, Través PG, Martín-Sanz P, Boscá L (2004). Simultaneous abrogation of NOS-2 and COX-2 activities is lethal in partially hepatectomised mice. J Hepatol.

[20] Zeini M, Hortelano S, Través PG, Gómez-Valadés AG, Pujol A, Perales JC (2005). Assessment of a dual regulatory role for NO in liver regeneration after partial hepatectomy: protection against apoptosis and retardation of hepatocyte proliferation. FASEB J.

[21] García-Trevijano ER, Martínez-Chantar ML, Latasa MU, Mato JM, Avila MA (2002). NO sensitizes rat hepatocytes to proliferation by modifying S-adenosylmethionine levels. Gastroenterology.

[22] Zeeh J, Platt D (2002). The aging liver: structural and functional changes and their consequences for drug treatment in old age. Gerontology.

[23] Anantharaju A, Feller A, Chedid A (2002). Aging liver.A review. Gerontology.

[24] Schmucker DL (1998). Aging and the liver: an update. J Gerontol A Biol Sci Med Sci.

[25] Ijtsma AJ, Boevé LM, van der Hilst CS, de Boer MT, de Jong KP, Peeters PM (2008). The survival paradox of elderly patients after major liver resections. J Gastrointest Surg.

[26] Tabernero A, Nadaud S, Corman B, Atkinson J, Capdeville-Atkinson C (2000). Effects of chronic and acute aminoguanidine treatment on tail artery vasomotion in ageing rats. Br J Pharmacol.

[27] Chou TC, Yen MH, Li CY, Ding YA (1998). Alterations of nitric oxide synthase expression with aging and hypertension in rats. Hypertension.

[28] Laubach VE, Shesely EG, Smithies O, Sherman PA (1995). Mice lacking inducible nitric oxide synthase are not resistant to lipopolysaccharide-induced death. Proc Natl Acad Sci USA.

[29] Mitchell C, Willenbring H (2008). A reproducible and well-tolerated method for 2/3 partial hepatectomy in mice. Nat Protoc.

[30] Livak KJ, Schmittgen TD (2001). Analysis of relative gene expression data using real-time quantitative PCR and the 2(-Delta Delta C(T)) Method. Methods.

[31] Zhang S, Chung WC, Wu G, Egan SE, Miele L, Xu K (2015). Manic fringe promotes a claudin-low breast cancer phenotype through notch-mediated PIK3CG induction. Cancer Res.

[32] Kohjima M, Tsai TH, Tackett BC, Thevananther S, Li L, Chang BH (2013). Delayed liver regeneration after partial hepatectomy in adipose differentiation related protein-null mice. J Hepatol.

[33] Wang X, Kiyokawa H, Dennewitz MB, Costa RH (2002). The Forkhead Box m1b transcription factor is essential for hepatocyte DNA replication and mitosis during mouse liver regeneration. Proc Natl Acad Sci USA.

[34] Morello D, Lavenu A, Babinet C (1990). Differential regulation and expression of jun, c-fos and c-myc proto-oncogenes during mouse liver regeneration and after inhibition of protein synthesis. Oncogene.

[35] Michalopoulos GK (2010). Liver regeneration after partial hepatectomy: critical analysis of mechanistic dilemmas. Am J Pathol.

[36] Yin S, Wang H, Park O, Wei W, Shen J, Gao B (2011). Enhanced liver regeneration in IL-10-deficient mice after partial hepatectomy via stimulating inflammatory response and activating hepatocyte STAT3. Am J Pathol.

[37] Liu Y, Shao M, Wu Y, Yan C, Jiang S, Liu J (2015). Role for the endoplasmic reticulum stress sensor IRE1α in liver regenerative responses. J Hepatol.

[38] Xiong Y, Connolly T, Futcher B, Beach D (1991). Human D-type cyclin. Cell.

[39] Guttridge DC, Albanese C, Reuther JY, Pestell RG, Baldwin AS Jr (1999). NF-kappaB controls cell growth and differentiation through transcriptional regulation of cyclin D1. Mol Cell Biol.

[40] Seki E, Brenner DA, Karin M (2012). A liver full of JNK: signaling in regulation of cell function and disease pathogenesis, and clinical approaches. Gastroenterology.

[41] Schwabe RF, Bradham CA, Uehara T, Hatano E, Bennett BL, Schoonhoven R (2003). c-Jun-N-terminal kinase drives cyclin D1 expression and proliferation during liver regeneration. Hepatology.

[42] Schaefer FM, Peng J, Hu W, Drvarov O, Nevzorova YA, Zhao G (2015). Bone marrow-derived c-jun N-terminal kinase- 1 (JNK1) mediates liver regeneration. Biochim Biophys Acta.

[43] Lim CP, Cao X (1999). Serine phosphorylation and negative regulation of Stat3 by JNK. J Biol Chem.

[44] Hortelano S, Dewez B, Genaro AM, Díaz-Guerra MJ, Boscá L (1995). Nitric oxide is released in regenerating liver after partial hepatectomy. Hepatology.

[45] Ballard RA, Truog WE, Cnaan A, Martin RJ, Ballard PL, Merrill JD (2006). Inhaled nitric oxide in preterm infants undergoing mechanical ventilation. N Engl J Med.

